# Supplementary material for: Extrinsic mechanical forces mediate retrograde axon extension in a developing neuronal circuit
Source: Nat Commun. 2017 Aug 17;8:282. doi: 10.1038/s41467-017-00283-3 (PMC5561127; doi:10.1038/s41467-017-00283-3)
Supplement: Supplementary file 1 — Supplementary Information [file 41467_2017_283_MOESM1_ESM.pdf]

File Name: Supplementary Information
Description: Supplementary Figures.

File Name: Supplementary Movie 1
Description: **Live imaging of OP morphogenesis upon blocking of cell proliferation with HUA treatment.** Live imaging on a *ngn1:gfp* embryo injected with H2B-RFP (magenta) to label cell nuclei, and treated with HUA from 12s to 24s. On the first frame, the dotted line indicates the brain surface. In the HUA condition, the nuclei are bigger than in untreated controls at 24s (end of the movie), as shown on the two pictures on the right, which shows efficient blocking of cell division (see also Supplementary Figure 3k). OP morphogenesis occurs normally in this condition. XY dorsal view, maximum projection, scale bar: 25 µm.

File Name: Supplementary Movie 2
Description: **Live imaging of OP morphogenesis.** Live imaging on a *ngn1:gfp* embryo between 12s and 24s, showing the coalescence of the two stripes of GFP+ OP cell domains into compact and spherical placodes on each side of the brain. On the last frame, the dotted line indicates the brain surface and asterisks show GFP expression in the brain. XY dorsal view, maximum projection of a 92 µm stack, scale bar: 50 µm.

File Name: Supplementary Movie 3
Description: **3D manual tracking of an individual OP cell.** Live imaging on a *ngn1:gfp* embryo injected with H2B-RFP (magenta) to label cell nuclei. The movie illustrates the 3D manual tracking of the nucleus of an anterior OP cell throughout OP morphogenesis, from 12s to 24s. The cell moved in the Z direction over time; for each time point we show the Z-section containing its nucleus. On the first and last frames, the number of the Z-section is shown ($\Delta Z = 2$ µm). The cell moved from the Z-section 45 to 38 and thus travelled 14 µm in the Z direction. The dotted line indicates the brain surface. XY dorsal view, scale bar: 25 µm.

File Name: Supplementary Movie 4
Description: **Live imaging of microtubules dynamics.** Live imaging on a wild type embryo transplanted with Doublecortin-GFP-expressing cells from 18s to 26 hpf. Note the presence of microtubules in the shaft of the protrusions and around cell bodies in OP cells during lateral movements. On the first and last frames, dotted lines indicate the brain surface. XY dorsal view, maximum projection of a 28 µm stack, scale bar: 25 µm.

File Name: Supplementary Movie 5
Description: **Long-term live imaging of protrusions.** Live imaging on an embryo injected with mbCherry (green) mRNA, from 18s to 26 hpf stages. A few OP cells express higher levels of mbCherry, which allows to visualise the retrograde elongation of their protrusions during lateral movements (white arrowheads), and their entry into the brain territory (red arrowhead). On the first and last frames, dotted lines indicate the brain surface. XY dorsal view, maximum projection of a 23 µm stack, scale bar: 25 µm.

File Name: Supplementary Movie 6
Description: **Live imaging of OP morphogenesis upon blocking of myosin II function.** Live imaging on *ngn1:gfp* embryos treated with Blebbistatin or Rockout from 12s to 24s, compared with an untreated control. On the first frames, dotted lines indicate the brain surface. On the last frames, red lines surround the OPs, showing longer and thinner placodes in both drug conditions. XY dorsal view, maximum projections, scale bar: 20 µm.

File Name: Supplementary Movie 7
Description: **Myosin II dynamics during convergence movements.** Live imaging on a wild type embryo transplanted with cells from a donor expressing the *ßactin:myosinII-GFP* transgene and mbCherry. The movie starts at 18s. Anterior OP cells undergoing convergence movements along the brain exhibit dynamic accumlations of myosin II in the front or back of their cell bodies. White arrows indicate instances of these myosin II accumulations. The yellow arrow shows the actomyosin ring in a dividing cell. XY dorsal view, maximum projection of a 44 µm stack, scale bar: 10 µm.

File Name: Supplementary Movie 8
Description: **Myosin II dynamics during lateral movements.** Live imaging on a wild type embryo transplanted with cells from a *ßactin:myosinII-GFP* transgenic donor injected with mbCherry mRNA. The movie starts at 18s and shows central OP cells undergoing lateral movements. Myosin II can not be detected in the cell bodies, but accumulates at the tip of the axonal protrusions (white arrows). Axons are indicated by green arrows. XY dorsal view, maximum projection of a 44 µm stack, scale bar: 10 µm.

File Name: Supplementary Movie 9
Description: **Behaviour of mosaic DN Rac-expressing cells.** Live imaging on a *ngn1:gfp* embryo transplanted with cells from a *ngn1:gfp* donor embryo coexpressing H2B-RFP (magenta) and DN Rac. On the first frame the dotted line indicates the brain surface. White arrows show DN-Rac expressing cells with impaired convergence movements, as compared with wild type neighbour cells (green arrow) that converge normally. XY dorsal view, maximum projection, scale bar: 20 µm.

File Name: Supplementary Movie 10
Description: **Actin dynamics during convergence movements.** Live imaging on a wild type embryo transplanted with cells from a donor embryo expressing Utrophin-GFP and mbCherry. Anterior OP cells converging along the brain exhibit dynamic filopodia and actin accumulation at their leading edge, two hallmarks of actively migrating cells. Green arrows indicate protrusions that do not belong to the two cells of interest, but to a more anterior cell following them. XY dorsal view, maximum projection of a 20 µm stack, scale bar: 10 µm.

File Name: Supplementary Movie 11
Description: **Actin dynamics during lateral movements.** Live imaging on a wild type embryo transplanted with cells from a donor embryo expressing Utrophin-GFP and mbCherry. In cells moving laterally, filopodia and actin are observed at the tip of axons, but not at the level of the cell bodies. Axons are indicated with green arrows. On the first frame, the dotted line indicates the brain surface. XY dorsal view, maximum projection of a 20 µm stack, scale bar: 10 µm.

File Name: Supplementary Movie 12
Description: **Laser ablation of an axon during lateral movement of the cell body.** Movie performed on an embryo expressing mosaic *ngn1:gfp* after laser ablation of an axon at 20s. The magenta cell track corresponds the cell with the ablated axon, whereas white tracks follow three neighbouring cells. On the first and last time frames, the dotted line indicates the brain surface, and magenta arrowheads point to the tip of the ablated axon that regrows towards the brain surface. XY dorsal view, maximum projection, scale bar: 20 µm.

File Name: Supplementary Movie 13
Description: **Live imaging on colcemid-treated embryos.** Movie performed on an embryo expressing mosaic *ngn1:gfp* and treated with colcemid from 12s to 24s. Yellow tracks show the convergence of two anterior cells, and magenta tracks show the lateral movements of round, axonless cells. On the first and last time frames, the dotted line indicates the brain surface. XY dorsal view, maximum projection, scale bar: 20 µm

File Name: Supplementary Movie 14
Description: **Nuclei rounding upon laser ablation of surrounding cells.** Two representative examples of laser ablation of cells surrounding elongated nuclei in the OP centre at 16s. On the first frames, the dotted lines indicate the brain surface, and the full lines shows the ablated region (ablation occurs at the 5s time point). Note that the elongated nuclei immediately change their shape after ablation of surrounding cells to acquire a rounder morphology. 1 Z-section, scale bar: 10 µm.

File Name: Supplementary Movie 15
Description: **Nuclei deformation and relaxation during lateral movements.** Movie performed on a *ngn1:gfp* embryo injected with H2B-RFP mRNA, labelling cell nuclei in red. In the centre of the placode, cell nuclei are initially highly deformed and elongated along the ML axis. Colored dots track two cells undergoing lateral movements. Their nuclei retrieve a round morphology right after their lateral departure. On the first and last frames, dotted lines indicate the brain surface. XY dorsal view, 1 Z-section, scale bar: 5 µm.

File Name: Supplementary Movie 16
Description: **Nuclei deformation and relaxation during lateral movements.** Movie performed on a *ngn1:gfp* embryo injected with H2B-RFP mRNA, labelling cell nuclei in red. The dot tracks a cell moving laterally and extending its axon, shown with a green arrow on the last frame. The cell nucleus is initially elongated along the ML axis and retrieves a round shape after lateral departure. On the first and last frames, dotted lines indicate the brain surface. XY dorsal view, 1 Z-section, scale bar: 5 µm.

File Name: Supplementary Movie 17
Description: **Laser ablation of intercellular contacts.** Representative examples of laser ablation of cell/cell interfaces in the OP centre at 16s, oriented perpendicular (left) and parallel (right) to the brain surface. The perpendicular cell/cell contact is more tensed than the parallel one, as shown by colored bars representing the vertex-vertex distances before (blue) and immediately after (yellow) the ablation. Green: *ngn1:gfp*, magenta: mbCherry. XY dorsal view, 1 Z-section, scale bar: 5 µm.

File Name: Peer Review File
Description:

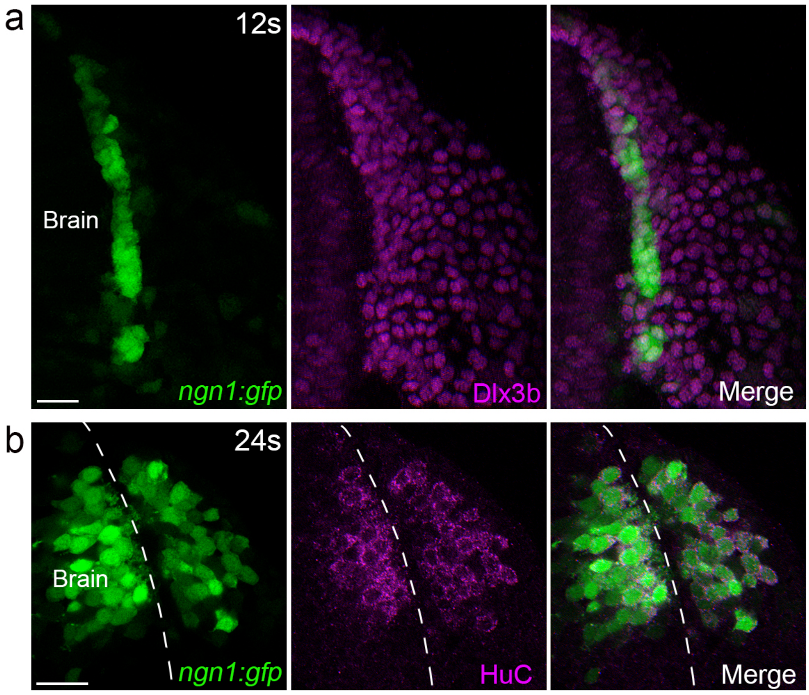

Supplementary Figure 1

Supplementary Figure 1. Characterisation of the *ngn1:gfp* transgenic line in the head during OP coalescence. (a) Dlx3b immunostaining performed on a ngn1:gfp embryo at 11-12s. GFP (green) is expressed in a narrow cell domain flanking the anterior brain and expressing Dlx3b (magenta), a marker for OP cells. (b) HuC immunostaining (magenta) performed on a ngn1:gfp embryo at 24s, showing co-localisation between GFP and HuC. Scale bars: 25 μm.

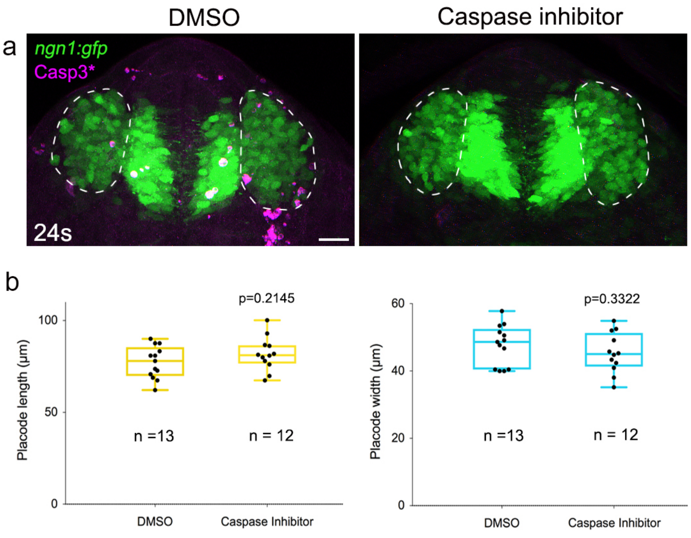

Supplementary Figure 2

Supplementary Figure 2. Apoptosis is not involved in OP morphogenesis.
(a) *ngn1:gfp* embryos incubated with a pan-caspase inhibitor (right) or DMSO (left) from 12s to 24s, stained with an anti-activated Caspase3 antibody (magenta). Dotted lines surround the two paired OPs. No morphogenesis phenotype can be detected upon apoptosis inhibitor treatment. (b) Quantification of OP length and width at 24s in embryos treated with the caspase inhibitor or DMSO controls. n indicates the number of analysed placodes (1 placode per embryo) in each condition. p values: unpaired two-tailed t-tests. Scale bar: 25 µm.

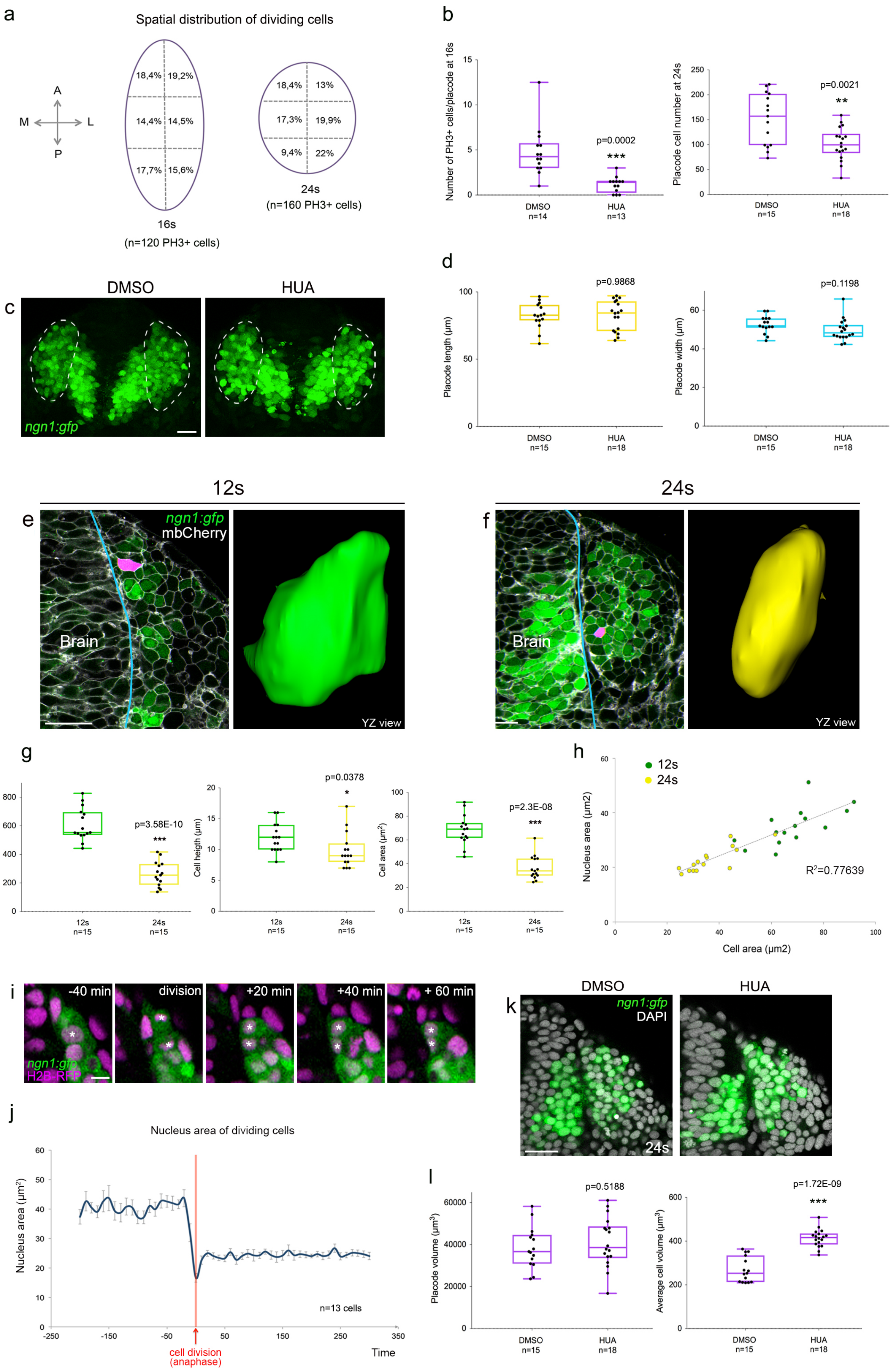

Supplementary Figure 3

Supplementary Figure 3. Proliferation and associated cell size changes are not involved in OP morphogenesis.
(a) Spatial distribution of PH3+ dividing cells in *ngn1:gfp* placodes at 16s and 24s. Comparison with a uniform distribution using a Chi2 test gave a p value>0.05, showing the uniform distribution of dividing cells. (b) Graphs showing the decrease in PH3+ cells at 16s, and the decrease in GFP+ cell numbers in OPs at 24s observed in embryos treated with the anti-proliferation drugs hydroxyurea and aphidicolin (HUA), or DMSO. (c) *ngn1:gfp* embryos incubated with HUA or DMSO from 12s to 24s, showing no overt OP morphogenesis defects upon HUA treatment. Dotted lines surround the two paired OPs. (d) Quantification of OP length and width at 24s in embryos treated with HUA or DMSO controls. In (b) and (c) n indicates the number of analysed placodes in each condition. p values: unpaired two-tailed t-tests. (e,f) *ngn1:gfp* embryos injected with mbCherry mRNA were fixed and stained with DAPI at 12s and 24s. Individual cell bodies were reconstructed in 3D using the mbCherry staining. Right panels show examples of reconstructed cells, corresponding to the cells highlighted in magenta in the left panels. (g) The 3D cell reconstruction allowed to quantify cell volume, cell height (along the DV axis) and cell area (maximum XY area) at 12s (n=15 cells from N=6 placodes) and 24s (n=15 from N=4 placodes). p values: unpaired two-tailed t-tests. Cell volume is smaller at 24s than at 12s, which is mostly due to a smaller cell size in the XY plan, rather than a reduction of cell height in the DV (Z) axis. (h) Nucleus area correlates with cell area, and can thus be used as a readout for cell size. (i) Instance of a placodal cell undergoing division, showing the decreased area in the two daughter cells, compared with the mother cell. (j) Dynamic measurement of areas of placode cell nuclei in live imaging experiments, showing that cell division coincides with a reduction in the area of nuclei (averaged on n=13 cells). (k) DAPI staining on 24s *ngn1:gfp* embryos incubated with HUA (rigth) or DMSO (left), showing placodes with similar size but bigger cells in HUA-treated embryos. (l) Quantification of OP volume and average cell volumes in embryos treated with HUA or DMSO. n indicates the number of analysed placodes in each condition p values: unpaired two-tailed t-tests. Scale bars: 25 μm except in I: 5 μm.

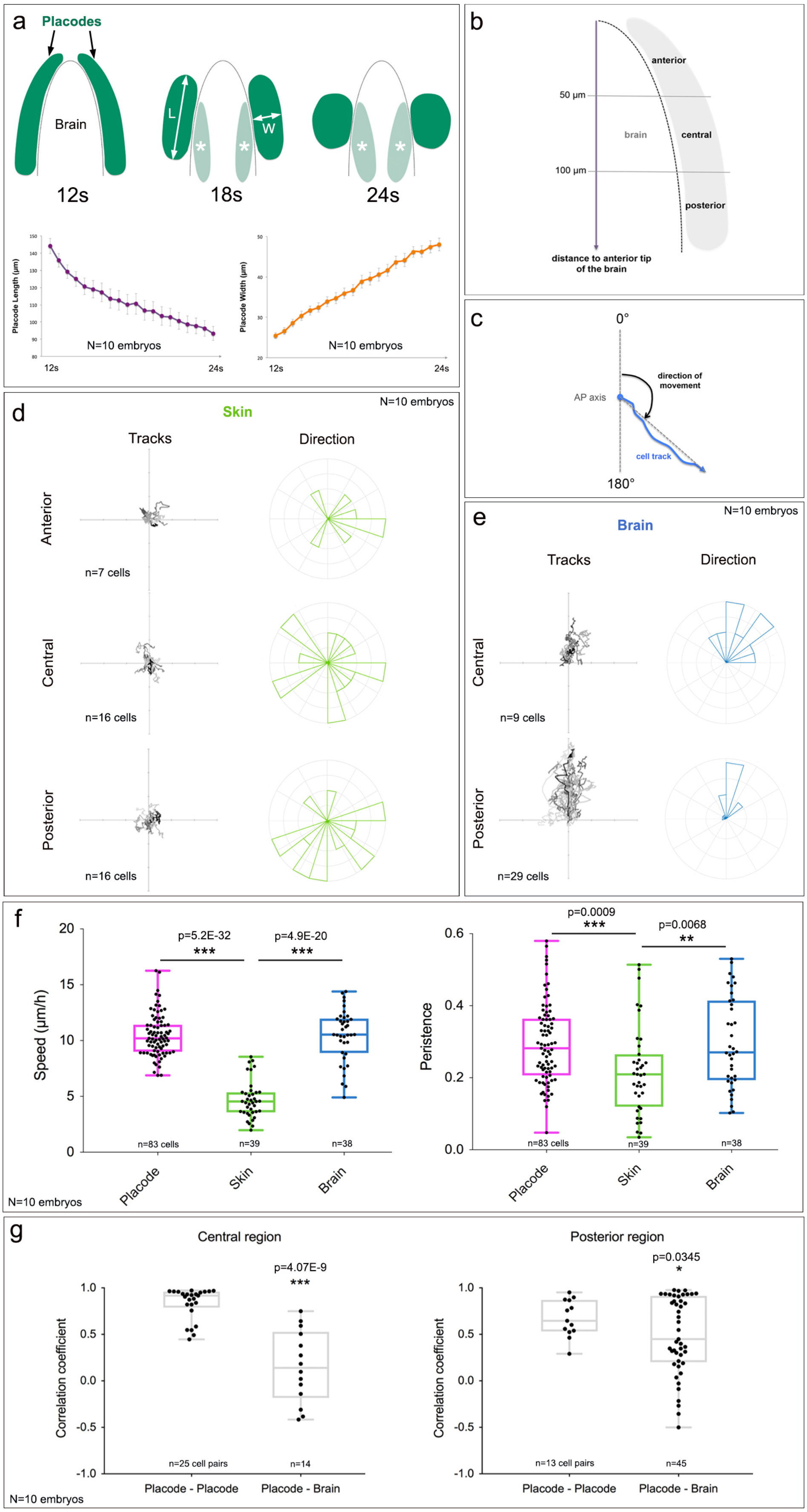

Supplementary Figure 4

Supplementary Figure 4. Additional live imaging results.
(a) Schematic view of OP coalescence visualised in ngn1:gfp embryos. Tissue-scale deformations during OP assembly: OP domains shrink along the AP axis (their length decreases, left graph) and get larger along the ML axis (their width increases, right graph). White asterisks indicate *ngn1:gfp+* cells in the brain. (b) OP cells are classified into anterior, central and posterior categories based on the distance between their initial position and the anterior tip of the brain. (c) The orientation of the movement is defined as the angle between the cell track and the AP axis. (d,e) Tracks of anterior, central and posterior cells, merged at their origin, and associated directions of movement, for skin (d) and brain (e) cells. Anterior brain cells cannot be tracked because they do not express *ngn1:gfp*. (f) Speed and straightness of the trajectories for placode, skin and brain cells. (g) Analysis of the correlation between cell trajectories in the central (left) and posterior (right) regions. Each dot represents the correlation between 2 given cell tracks from the same movie. We analysed pairs of placode cells (placode-placode) or pairs with 1 placode cell and 1 brain cell (placode-brain). p values: unpaired two-tailed t-tests.

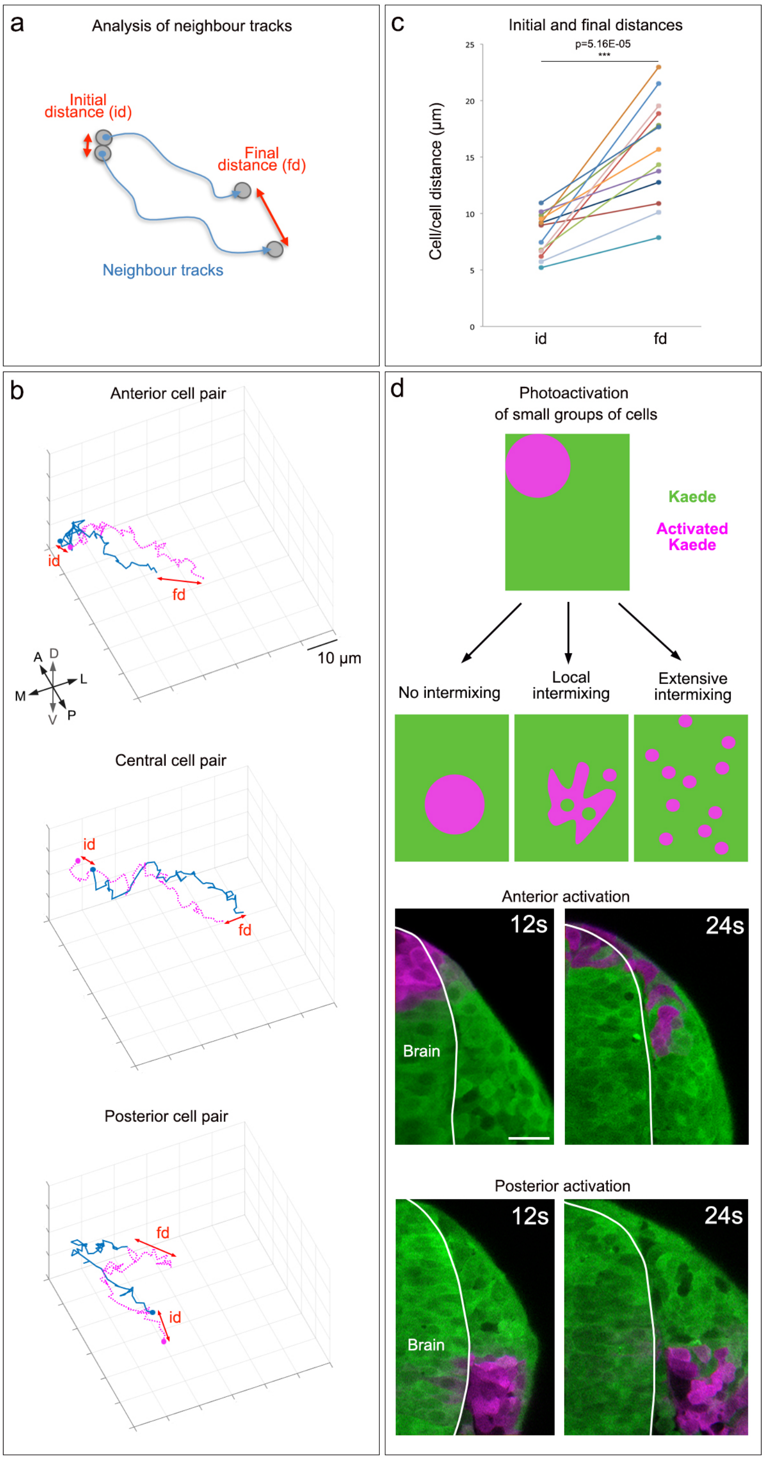

Supplementary Figure 5

Supplementary Figure 5. Analysis of neighbour exchange during OP morphogenesis.
(a) To assess whether cells exchange neighbours during OP morphogenesis, we analysed the tracks of cell neighbours, and measured the initial distance (id, at 12s) and the final distance (fd, at 24s) separating the two cell centres. (b) Examples of 3D trajectories for anterior, central and posterior pairs of cells, showing an overall coordination of their movements, with a small increase in the distance separating their centres. (c) Quantification of initial and final distances between cell pairs (n=13 cell pairs) shows that the distance is doubled during morphogenesis, suggesting short-range cell intermixing. p value: two-tailed paired t-test. (d) Photoactivation of Kaede in small groups of placodal cells at 12s results in a local intermingling of green (not photoactivated) and magenta (photoactivated) cells at 24s. Scale bar: 25 µm.

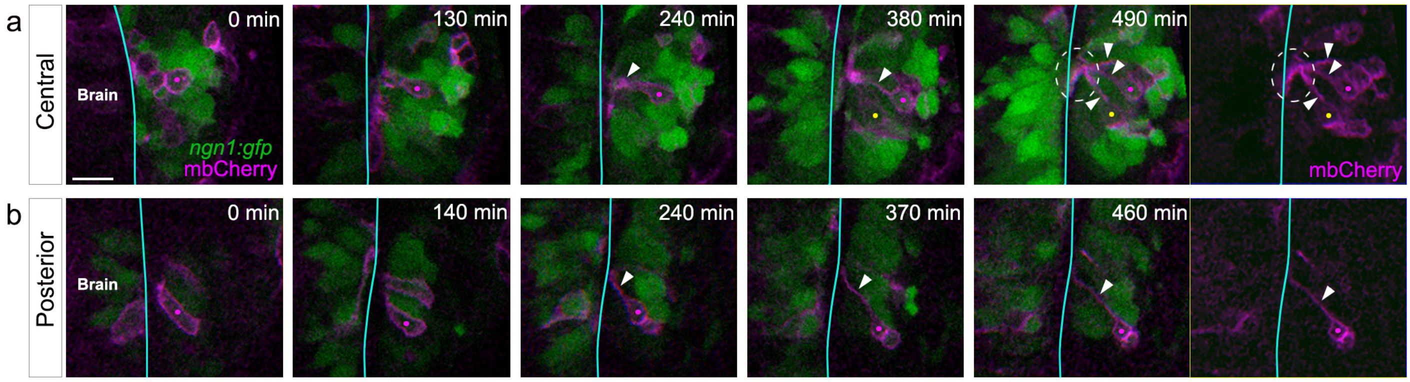

Supplementary Figure 6

Supplementary Figure 6. Morphologies of placodal cells expressing low levels of GFP.
(a,b) Two time lapse sequences showing instances of central (a) and posterior (b) low *ngn1:gfp+* cells undergoing lateral movements. Cells of interest are indicated with pink and yellow dots. The brain surface is indicated by a blue line. While moving laterally, the cells (labelled with mosaic mbCherry) extend a long cytoplasmic process contacting the brain surface (white arrowheads), as shown for high GFP-expressing cells in Figure 2. On the up right panel, note that all the protrusions of mbCherry-labelled placode cells meet to form a bundle on the brain surface (white dotted line). Scale bar: 25 µm.

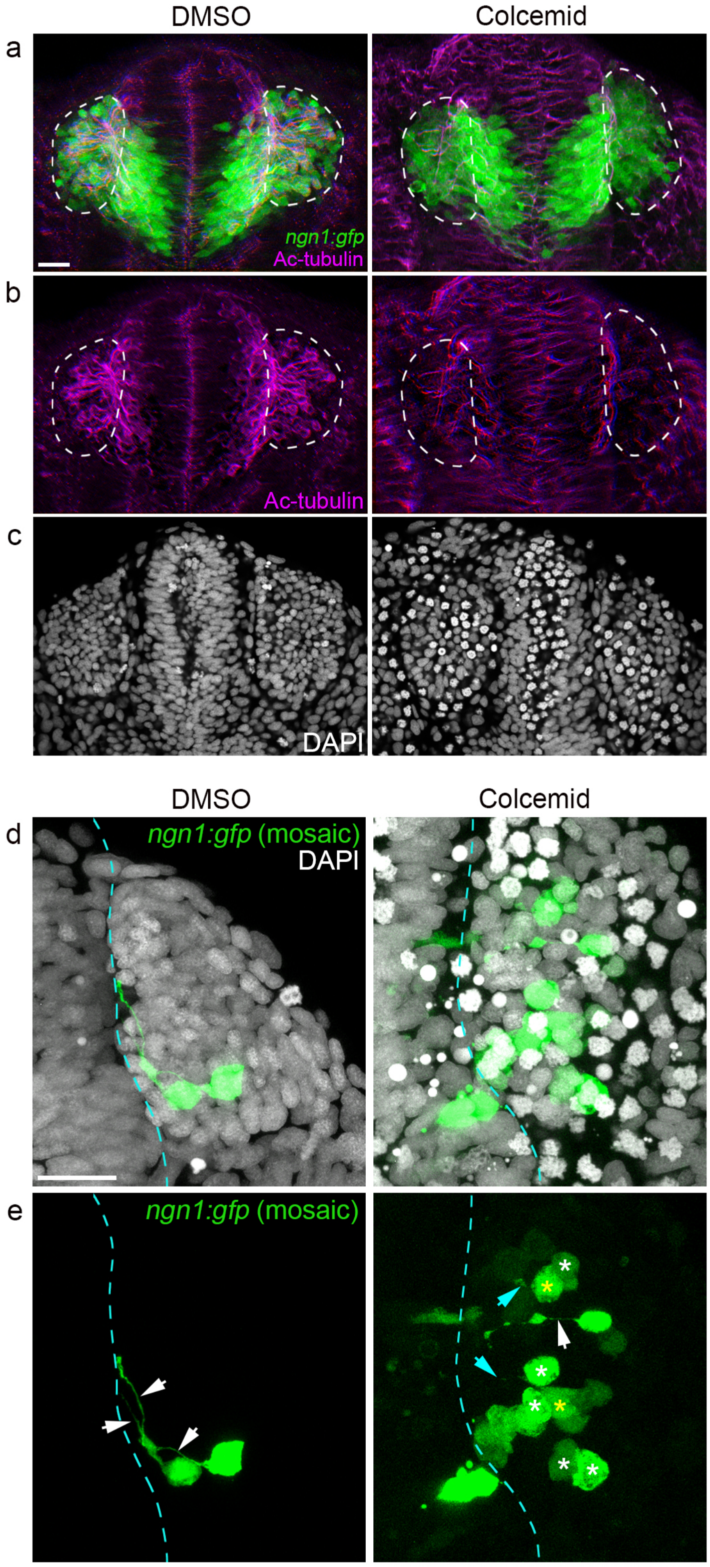

Supplementary Figure 7

Supplementary Figure 7. Additional results for colcemid treatment experiments.
(a,b) *ngn1:gfp* embryos treated with colcemid or DMSO, and stained for acetylated tubulin at 24s. (a) shows the merge picture, and (b) the acetylated tubulin staining only. The acetylated tubulin appears affected and disorganised in the head of colcemid-treated embryos, and is strongly reduced in OPs (surrounded by white dotted lines). (c) XY sections of DAPI staining of the embryos shown in (a) and (b). Note the numerous nuclei blocked in mitosis in colcemid-treated embryos. These results confirm that colcemid treatment affects microtubule dynamics and metabolism. (d,e) Wild type embryos were injected with *ngn1:gfp* DNA to obtain a mosaic labelling of OP neurons, treated with colcemid or DMSO and stained for DAPI at 24s. In DMSO controls, mosaic labelling allows to visualise axonal protrusions connecting cell bodies to the brain surface (white arrows). In colcemid-treated embryos, some GFP+ cells possess a normal axonal protrusion (white arrow), but a significant proportion of GFP+ cells show shorter axons that do not contact the brain (yellow asterisks indicate the cell bodies of such cells, and blue arrows their short protrusions) or no axonal protrusion at all (white asterisks indicate their somata). Scale bars: 25 µm.

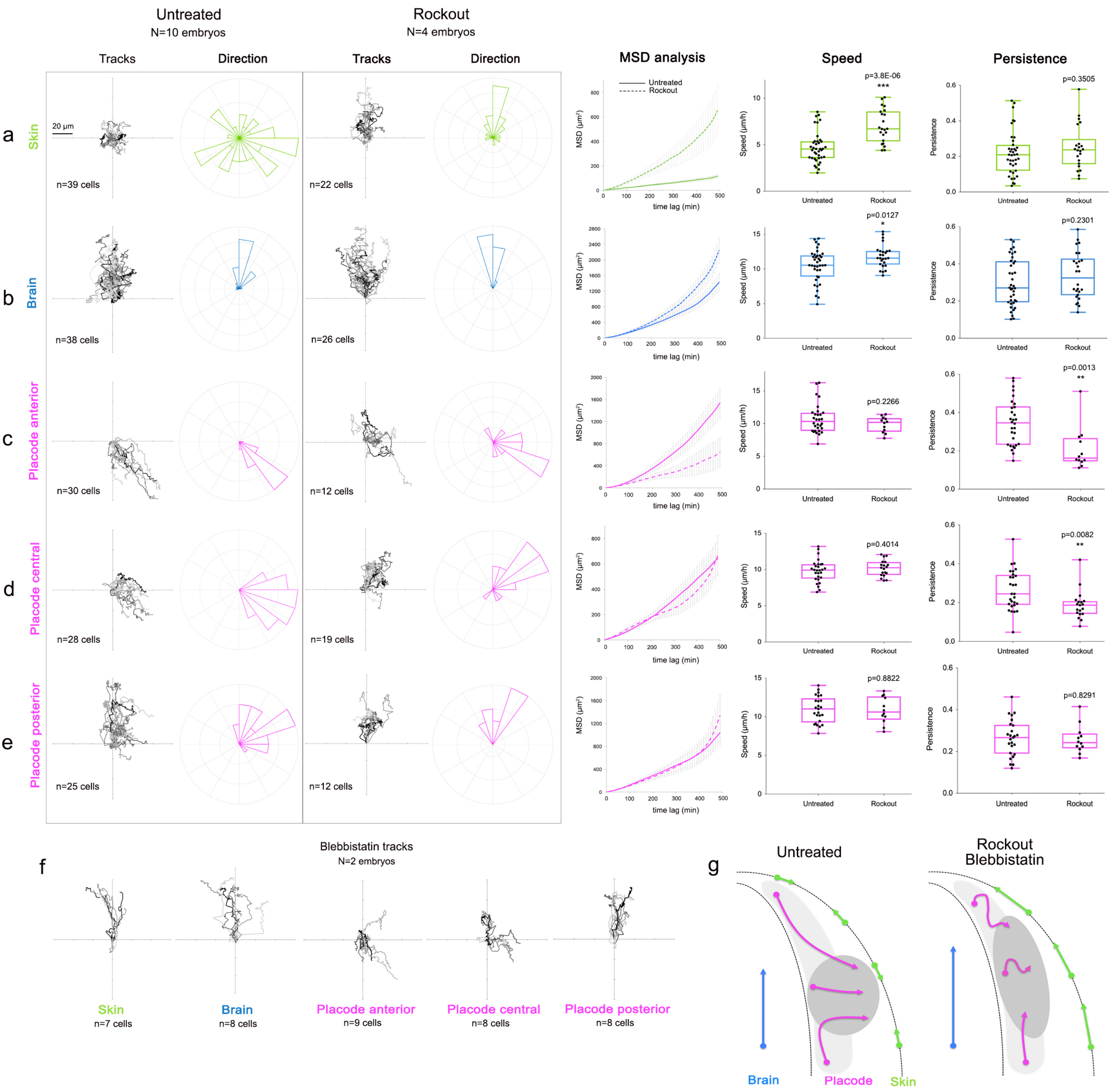

Supplementary Figure 8

Supplementary Figure 8. Quantitative live imaging analysis of cell movements upon global inhibition of myosin II activity. (a-e) Tracks of skin (a), brain (b), anterior placode (c), central placode (d) and posterior placode (e) cells, merged at their origins, and their associated direction of movement, MSD, speed and persistence analysis, from movies performed on Rockout-treated embryos (n=4 embryos) and untreated controls (n=10 embryos). p values: two-tailed unpaired t-test. Anterior and central cells show perturbation in direction and persistence of movement upon Rockout treatment. Brain and posterior placode cells are relatively unperturbed. Strikingly, skin cells show a clear anteriorward movement with increased speed, persistence and MSD in Rockout-treated embryos, which is clearly different from the unoriented diffusive-like behaviour in controls. f) Tracks of anterior, central and posterior placodal cells and of skin and brain cells, merged at their origins, from movies performed of blebbistatin-treated embryos (n=2 embryos). The perturbations of cell movements are similar in Blebbistatin and Rockout conditions. g) Summary of the movements in control (left) and drug (right) conditions. Grey regions represent the original and final shapes of the OP. Only the lateral boundary of the skin is shown but the skin epithelium overlies the whole brain and placode tissues.

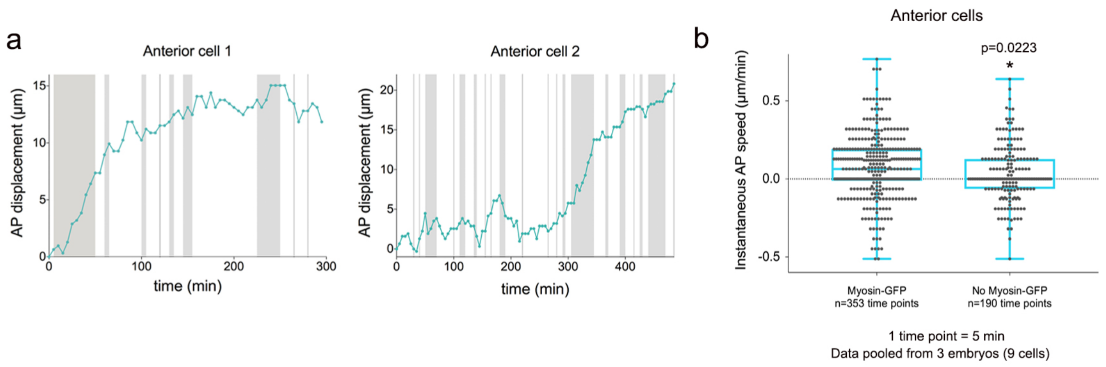

Supplementary Figure 9

Supplementary Figure 9. Correlation between anterioposterior OP cell movements and Myosin II-GFP dynamic accumulations.
(a) Graphs showing the extent of the correlation between the net displacement of cells during their AP convergence movements and the presence of Myosin II-GFP pulses (indicated by grey bars) in the front or rear of their cell body, in two representative anterior OP cells. (b) Graph representing the instantaneous AP speed of anteroposterior migrating cells in the presence (left) or absence (right) of Myosin II accumulation. p value: two-tailed unpaired t-test.

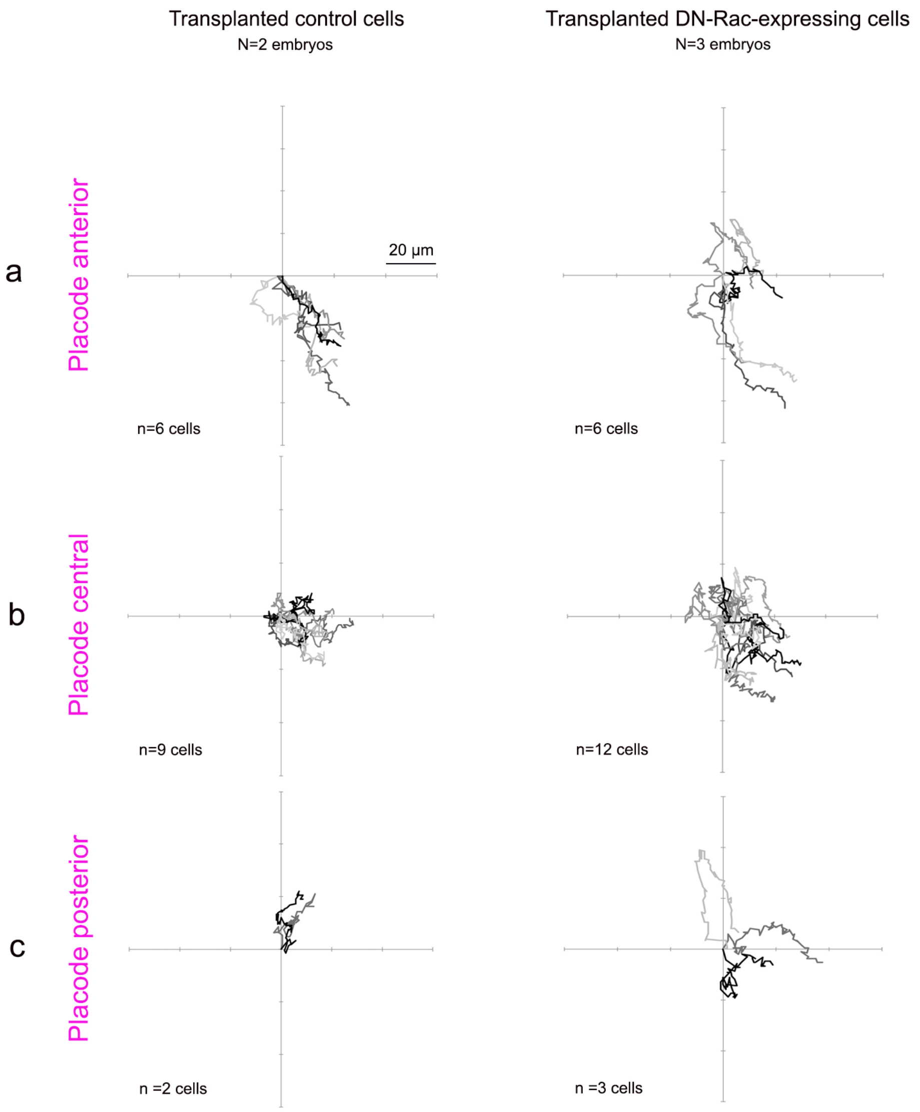

Supplementary Figure 10

Supplementary Figure 10. Live imaging analysis of cell movements upon mosaic expression of DN-Rac.
Cells from *ngn1:gfp* donor embryos co-expressing H2B-FRP and a dominant-negative form of of Rac (DN-Rac), or the H2B-CFP protein alone (controls) were transplanted into *ngn1:gfp* host embryos, and tracked over time. The figure shows tracks of anterior placode (a), central placode (b) and posterior placode (c) cells merged at their origins, for transplanted cells expressing DN-Rac, as compared with control transplanted cells.

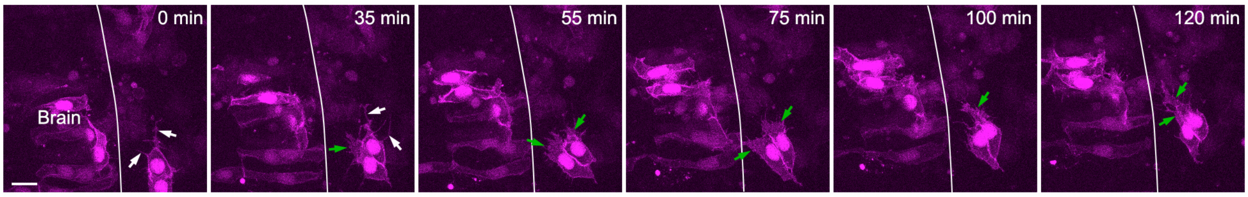

Supplementary Figure 11

Supplementary Figure 11. Pacodal cells exhibit filopodia and lamellipodia-like protrusions during convergence. Live imaging on a wild type embryo transplanted with mbCherry and H2B-RFP co-expressing cells, from 12s onwards. Note the filopodia in the front of posterior placodal cells converging towards the center (white arrows), but also the presence of large lamellipodia-like protrusions (green arrows). The white line indicates the brain surface. XY dorsal view, anterior to the top, scale bar: 5 µm.

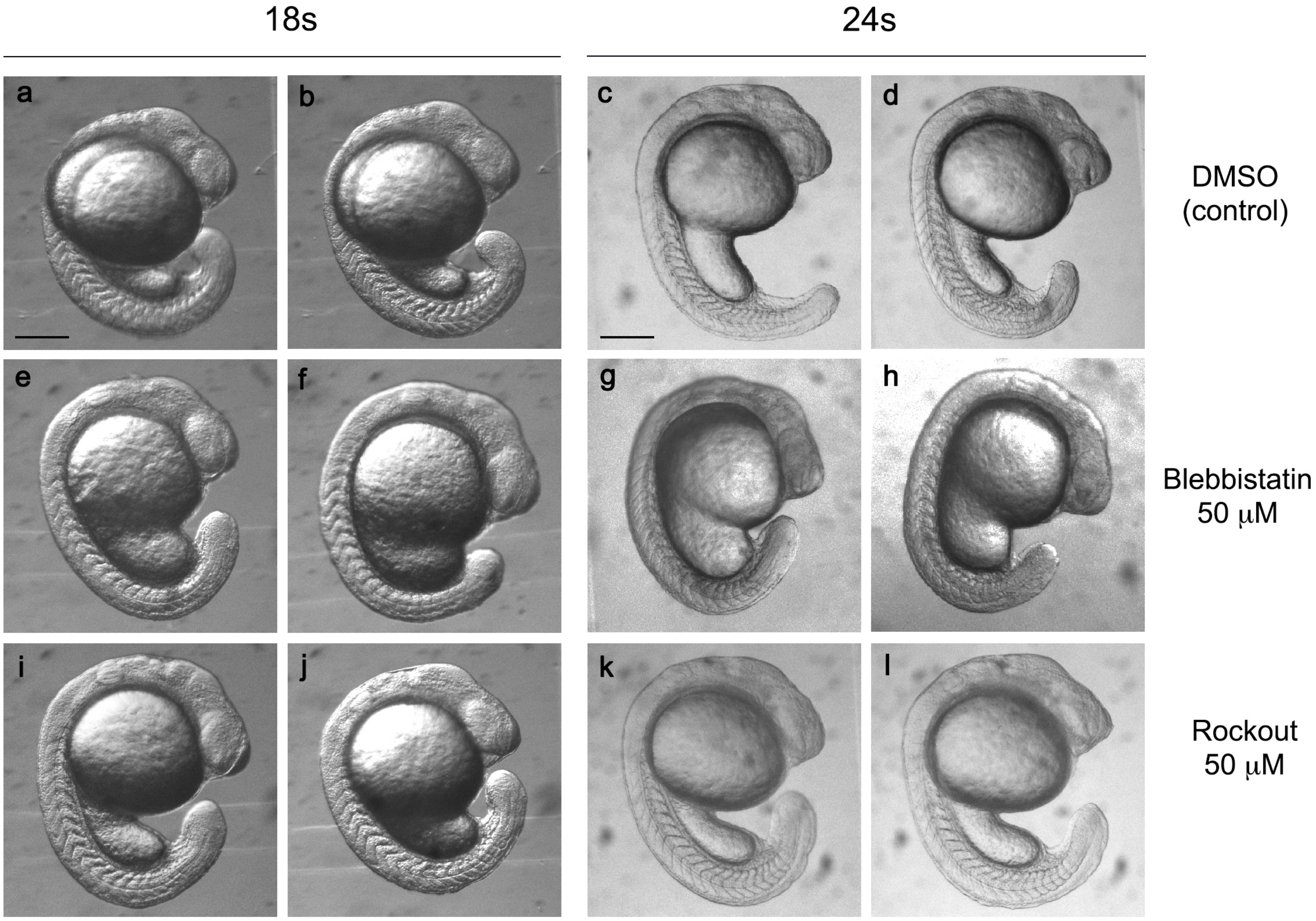

Supplementary Figure 12

Supplementary Figure 12. Blebbistatin and Rockout-treated embryos are alive and develop at normal speed. Zebrafish embryos incubated from the 12s stage with 50 μM blebbistatin (a-d), 50 μM Rockout (e-h), or DMSO (i-l) and let to develop until the 18s or the 24s stage. Both treatments led to a thickening of the yolk sac extension at 18s (arrowheads). This thickening persisted at 24s only in Blebbistatin-treated embryos. Overall embryo development was similar in all three conditions. Scale bars: 200 μm.
